# Fair concordance between Google Trends and Danish ornithologists in the assessment of temporal trends in Danish bird populations highlights the informational value of big data

**DOI:** 10.1007/s10661-024-12439-y

**Published:** 2024-02-16

**Authors:** Per M. Jensen, Finn Danielsen, Stine K. Jacobsen, Thomas Vikstrøm

**Affiliations:** 1 Department of Plant and Environmental Sciences, University of Copenhagen, Thorvaldsensvej 40, 1871 Frederiksberg, Denmark; 2 NORDECO, Skindergade 23, 1159 Copenhagen K, Denmark; 3 DOF/BirdLife Denmark, Vesterbrogade 140A, 1620 Copenhagen V, Denmark

**Keywords:** Google Trends, Validation, Bird species, Population trends

## Abstract

The ongoing depletion of natural systems and associated biodiversity decline is of growing international concern. Climate change is expected to exacerbate anthropogenic impacts on wild populations. The scale of impact on ecosystems and ecosystem services will be determined by the impact on a multitude of species and functional groups, which due to their biology and numbers are difficult to monitor. The IPCC has argued that surveillance or monitoring is critical and proposed that monitoring systems should be developed, which not only track developments but also function as “early warning systems.” Human populations are already generating large continuous datasets on multiple taxonomic groups through internet searches. These time series could in principle add substantially to current monitoring if they reflect true changes in the natural world. We here examined whether information on internet search frequencies delivered by the Danish population and captured by Google Trends (GT) appropriately informs on population trends in 106 common Danish bird species. We compared the internet search activity with independent equivalent population trend assessments from the Danish Ornithological Society (BirdLife Denmark/DOF). We find a fair concordance between the GT trends and the assessments by DOF. A substantial agreement can be obtained by omitting species without clear temporal trends. Our findings suggest that population trend proxies from internet search frequencies can be used to supplement existing wildlife population monitoring and to ask questions about an array of ecological phenomena, which potentially can be integrated into an early warning system for biodiversity under climate change.

## Introduction

The growing human population and the associated exploitation of natural resources have directly and indirectly affected many wildlife populations (Galli et al., 2014). Overexploitation and habitat loss were the main drivers of population declines in the past decades (Rosser & Mainka, 2002), while climate change is likely to be the main contributor in years to come (Román-Palacios & Wiens, 2020). The Intergovernmental Panel on Climate Change (IPCC) recently released its sixth report on *Climate Change; Impacts, Adaptation, and Vulnerability* (IPCC, 2022). The report repeatedly argues that surveillance or monitoring is critical and proposes that monitoring systems should be developed, which not only track developments but also function as “early warning systems,” such that negative effects can be ameliorated through timely interventions. The IPCC does not currently define objectives for “early warnings” for biological systems but generally implies that the systems should be able to detect small and essentially inconsequential changes, which in unambiguous terms forewarns that significant changes, such as species declines and extinctions, will occur later.

Relatively few attempts have been made to conceptualize or develop early warning systems for the slow and progressive loss of biodiversity associated with climate change (Antonelli et al., 2023; Barnard et al., 2017).

**Fig. 1:**
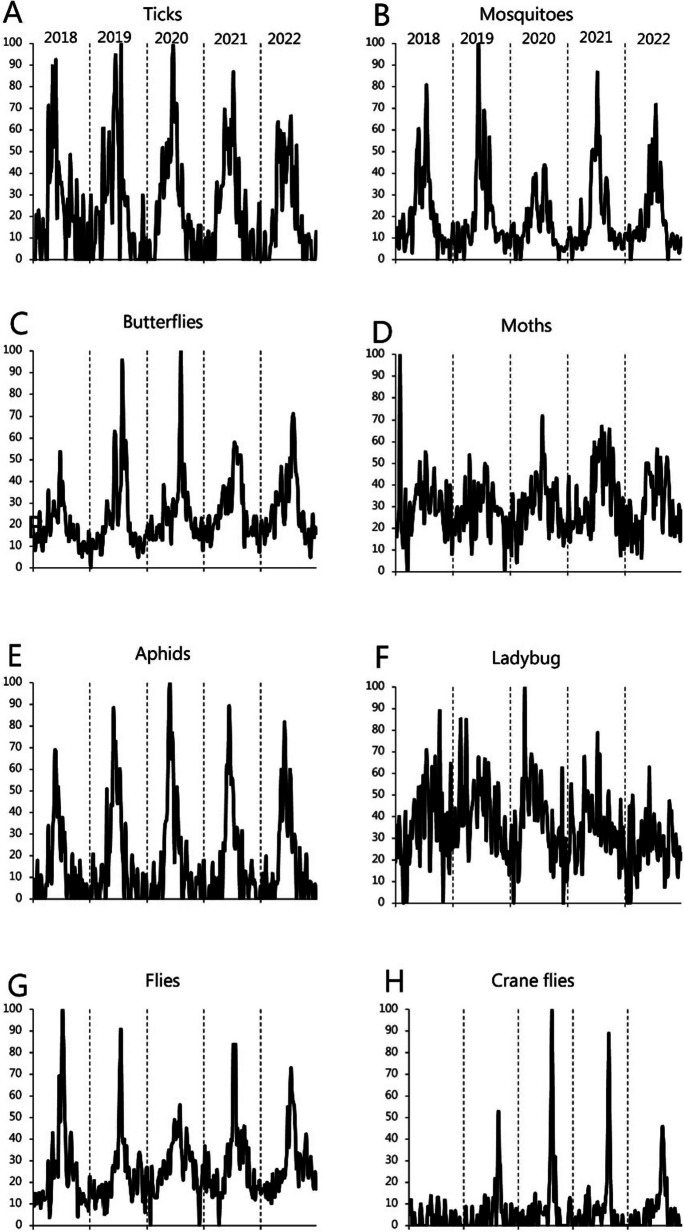
Examples of types of invertebrates that the Danes search for information on using Google. The data was retrieved from Google Trends using the Danish terms for **A** ticks (Danish: flåter), **B** mosquitoes (Danish: myg), **C** butterflies (Danish: sommerfugle), **D** moths (Danish: møl), **E** aphids (Danish: bladlus), **F** ladybug (Danish: mariehøne), **G** flies (Danish: fluer), and **H** crane flies (Danish: Stankelben). The given type of records can be subjected to analysis regarding seasonality, month of peak activity, etc. without other sources of data. The retrieval and import into Microsoft Excel took 15 min

Conventional monitoring mostly delivers population estimates for vertebrate species, which, due to the practicalities of monitoring, have limited accuracy (Krebs, 1999, Seber and Schofield, 2019). The accuracy is even poorer for the countless number of invertebrate species that provide critical functions in most ecosystems (Samways, 2019). Unfortunately, the choice of sampling method (Krebs, 1999, Seber and Schofield, 2019) and weather conditions (Nation, 2022) influences observations on invertebrates as much as their abundance. The monitoring is further complicated by diverse affiliations with a wide range of micro-environments and complex lifecycles that lead to differential dependencies on weather patterns and climate (Kingsolver et al., 2011). Ticks, fleas, butterflies, moths, aphids, ladybugs, flies, crane flies, earthworms, ants, etc. (Fig. 1) all qualify as potential sentinels for climate change, but the scientific community, governments, and NGOs rarely monitor invertebrate species unless they pose a threat to plant, animal, or human health (Severini et al., 2008; Vogelgesang et al., 2020). There is to our knowledge no system that systematically monitors and interprets subtle indices of change such as shift in behavior and phenology, which could serve as early warnings.

Citizen science has been suggested as a method for improving or supplementing classical monitoring schemes (Bonney, 2021; Hester & Cacho, 2017). Ideally, these methods deliver more data than conventional monitoring schemes, because they include larger groups of observers and a wider scope of environmental resources (Musvuugwa et al., 2021). The largest numbers of observers are found in passive monitoring programs where, e.g., internet search frequencies are used as indices of temporal variation in natural resources and wildlife populations (Jensen et al., 2022; Schuetz et al., 2015; Soulsbury, 2020). The Internet records are however not ideal for monitoring animal populations because the general population is unable to identify invertebrates at the species level, and hence, the data quality is often, by scientific standards, considered of poor accuracy and precision (Balázs et al., 2021). Citizen science and community monitoring are better appreciated for their community value (Danielsen et al., 2018; Lukyanenko et al., 2016), and wider informational scope (Jarić et al., 2020), which permits a broader assessment of ecological phenomena. Despite this, the informational value of passive monitoring schemes and big data remains largely unexplored. The progress is slow because investigations/validations require that the true information is known, that we can compare their observations with records from a group of observers that we trust, or that we by other methods can infer that big data provides credible information.

There are in a Danish context limited options for assessing the credibility of internet searches because few terrestrial animal populations are monitored. The only group of animals that are intensely monitored and rigorously analyzed is birds. The monitoring is undertaken by members of the Danish Ornithological Society (BirdLife Denmark/DOF), which frequently reviews and publishes its assessments (DOF, 2023). Previously, Mittermeier et al. (2019) identified short-term temporal correspondence between eBird sightings and Wikipedia page views for a sample of migratory birds in Italy, Germany, Sweden, and the United States, demonstrating that, for some migratory birds, the physical presence of the species in a region correlated with increased public interest.

More recently, Mittermeier et al. (2021) found a correlation between the frequency with which people have direct encounters with species in the wild and online interest in > 10,000 species of birds across multiple regions of the world. Internet searches will likely also inform on bird abundance in Denmark, but these numbers have little value as predictors of future abundance. If we seek to predict future challenges, we must as a minimum be able to show that internet searches deliver credible information on population trends/declines as these are associated with a risk of extinction.

We investigated the temporal trends in search frequencies for 106 Danish bird species as given by Google Trends (GT) in the period 2015 to 2020 and compared these trends with the most recent population trends provided by DOF. This is to our knowledge the first study that seeks to validate population trends inferred by big data.

## Methods and materials

Our study is designed as a comparison of two sets of observers that, under similar circumstances, monitor several bird populations (Landis & Koch, 1977). One set of observers (DOF) is considered the “reference method,” while the Google Trends (GT) data generated by the general Danish human population are considered to be the “new method.” We extracted the data to fit the data format published by DOF in its most recent atlas survey (DOF, 2022a), which allowed us to compare population developments in terms of “declining,” “no-change,” or “increasing” trends. The two sets of observers are unfortunately not perfectly matched as the GT data, referred to different years (2014–2017 vs. 2015–2021). The DOF trends further include a reference to earlier information (Atlas II: 1993–1996), while the trends for GT data were restricted to the given years and subjected to linear regression within the given period. We suspect that DOF members are more prone to use both visual and audial cues, than the general population. Last, DOF records are geographically representative, while GT data likely have a higher contribution from populated areas. These methodological differences will all reduce the agreement between observers, but similar trends are expected because the development in bird populations generally transpires over decades rather than years.

### The settings and observer base

Denmark is a small country in Northern Europe (latitude 54–57 N longitude 8–14 E). Exactly 500 bird species have been recorded since 1800 (Netfugl, 2022; Kjærbølling, 1852), but only approx. 200 are considered to be common. The Danish human population totaled 5.6 mill people and 2 mill households in 2015–2020 (Statistics Denmark, 2022). Most Danish households have computers (Statistica.com, 2022a), and the Google Internet browser is the most commonly used search engine (market share > 90% (Statistica.com, 2022b). DOF members (www.dof.dk) have been monitoring bird populations since 1906, and volunteers among the current approx. 17,500 members provide most of the information available on Danish bird populations. DOF members deliver their observations electronically into a database (www.dofbasen.dk). The number of records/observations in the database totaled 30,634,888 on December 1, 2022, and accrues with approx. 1 mill observations per year. While the entries in the electronic database are opportunistic/unsolicited, the associated atlas projects and others are planned and standardized (stratified) by scientific methodology (point counts, DOF 2022a). The Atlas III publication provided information on the abundance/range (given as the number of 5 × 5 km quadrats with “certain,” “probable,” or “possible” presence), and the associated population trends.

### GT data retrieval

Google Trends was chosen for data extraction because it provides easy access to the underlying database of internet search frequencies. It means that also biologists with limited insight into big data management can extract data, which is being presented as time series data. These time series can be perused and analyzed by conventional methods, permitting that temporal dynamics can be inspected for indications of phenological changes that precede population declines. Data is available for a wide range of taxons/terms (Fig. 1), which are used by the general public.

When search terms are entered in Google Trends (GT), the site engine automatically displays relative daily search frequencies for the last 12 months for the country, in which the computer is located (Google Trends, 2022). The search can then be modified to include other areas and timespan, just as a context can be assigned to the search. Context-specific terms are associated with a suffix, e.g., “bird”, which must be selected from the drop-down list. Comparisons between search terms are performed by adding another search term, in which case the output will include information on both. For comparisons, a bar chart appears with information on the “relative average frequency” of the given search term(s). This information can also be retrieved for a single term by just comparing the term to itself. We retrieved information on the relative GT abundance by entering the species name and selecting records on birds (suffix: birds). The search frequencies for bird species occurring in Denmark were then downloaded for the period 1/1 2011 to 31/12 2021 for species that were considered “common,” by either DOF range, number of breeding pairs, or GT search frequencies. This yielded 132 monthly observations for 129 species.

### Selection of time series and exclusion of data records

**Fig. 2:**
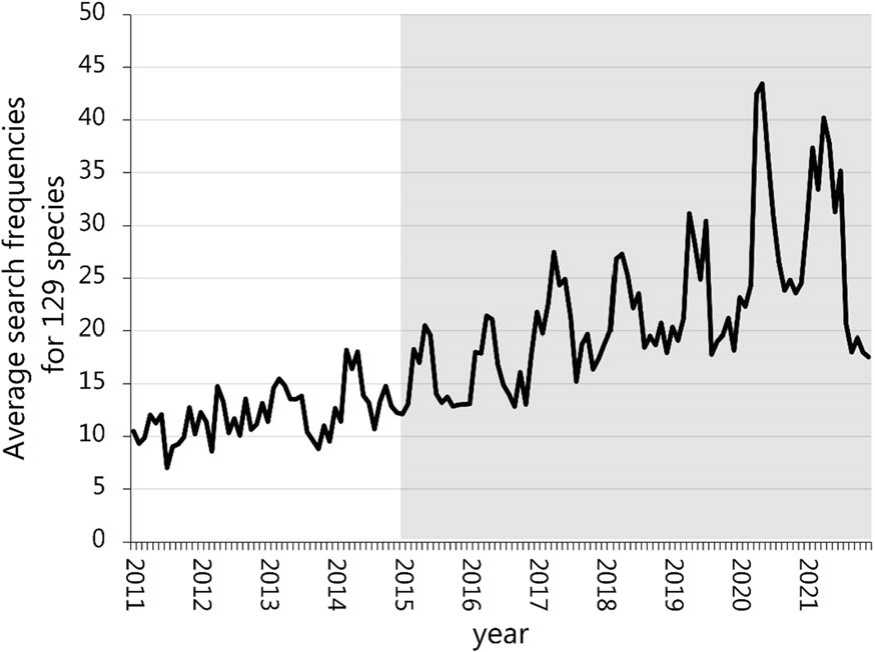
Monthly mean Google Trends search frequencies for 129 bird species for the period 2011 to 2021. The gray area marked the period included in this study

Google search frequencies are available from Google Trends from 2004. However, a perusal of the time series data suggested that part of the data for various reasons had to be excluded. First, the temporal dynamics for the early years did not reveal the expected seasonal variation (Fig. 2), and we therefore chose to base our assessment on the data from 1/1 2015 to 31/12 2021. Second, 21 species were excluded, as the Atlas III record (DOF 2022a) did not provide information on population trends. Last, we generated a clear-cut inclusion criterium, by removing two records with an average GT frequency of less than 15. A further deletion of species with search frequencies of less than 16 would reduce the number of species to 75 and negatively affect our ability to evaluate the records. The reduced dataset included 84 monthly records for 106 species.

### Statistical models

We used four different models to assess the temporal trend in the GT data. The first model (model 1) was a simple linear regression with search frequencies vs. time (running month). In the second, we expanded the model to include seasonal effects (month). Both models were executed as generalized linear models (Proc GLM, SAS 9.4) that provided an *R*^2^ as a goodness-of-fit measure. We had some concerns that the absolute trend in the GT data would be unsuitable for assessing population trends, due to a general increase in many species (Fig. 2). We therefore also assessed the relative contribution of each species to the total number of searches, in a model analog to models 1 and 2. Models 3 and 4 were executed under Proc Genmod (dist = bin, link = logit, SAS 9.4), which provided Akaike information criterion (AIC) as a goodness-of-fit measure.

The GT trends were first assessed by their association with the categorization provided by DOF (declining, no-change, or increasing; Atlas III). Mean GT trends were analyzed with DOF trend categories in a generalized model that included a paired *t* test subroutine (Proc GLM/ LSMEANS with Tukey adjustment, SAS 9.4).

We then converted the GT trends to categorical values by defining cutoff values that transformed low GT trend values into the categories: declining (− 1), no-change (0), and increasing (+ 1). The cutoff values were set to deliver a reasonable number in the no-change category. Last, the association between the DOF trend and GT trend categories was assessed by frequency analyses (Proc Freq, subroutine “Agree,” SAS 9.4), which provided a chi-sq and weighted kappa, i.e., a measure of agreement. Weighted kappa values from 0.0 to 0.2 were accepted as slight agreement, 0.21 to 0.40 as fair, 0.41 to 0.6 as moderate, 0.61–0.80 as substantial, and 0.81 to 0.99 as nearly perfect agreement (Landis & Koch, 1977). We close by exemplifying putative contributors to observer bias in the GTdata by removing certain species from the analyses according to whether they are large (wingspan > 100 cm, DOF, 2022b) and frequently appear in recipes in a Danish online cookbook (The best cookbook of all times, 2022).

## Results and discussion

**Fig. 3:**
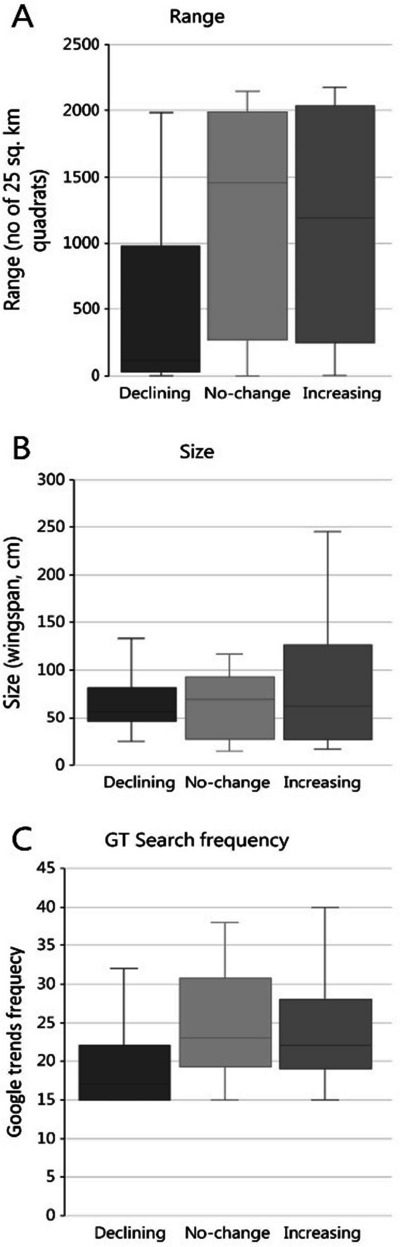
Range, size, and average search frequency for species that by Danish Birdlife was assessed to be in the declining, not-changing, or increasing category. **A** The range is given as the sum of certain, probable, and possiblepresence in 5 × 5 km^2^ quadrats (Atlas III). **B** The size refers to the wingspan in cm (DOFbasen), while **C** the search frequency refers to the count of specific GT search frequencies

The DOF assessment of the 106 species populations included 21 species that were in the declining, 21 in the not-changing, and 64 species in the increasing category. The species that were declining had generally smaller ranges (Fig. 3A), but the trend had no apparent association with their size (wingspan, Fig. 3B). The lower ranges (abundance) among species in decline were also noticeable in the GT search frequencies (Fig. 3C), indicating that search frequencies are lower for species with smaller ranges.

**Fig. 4:**
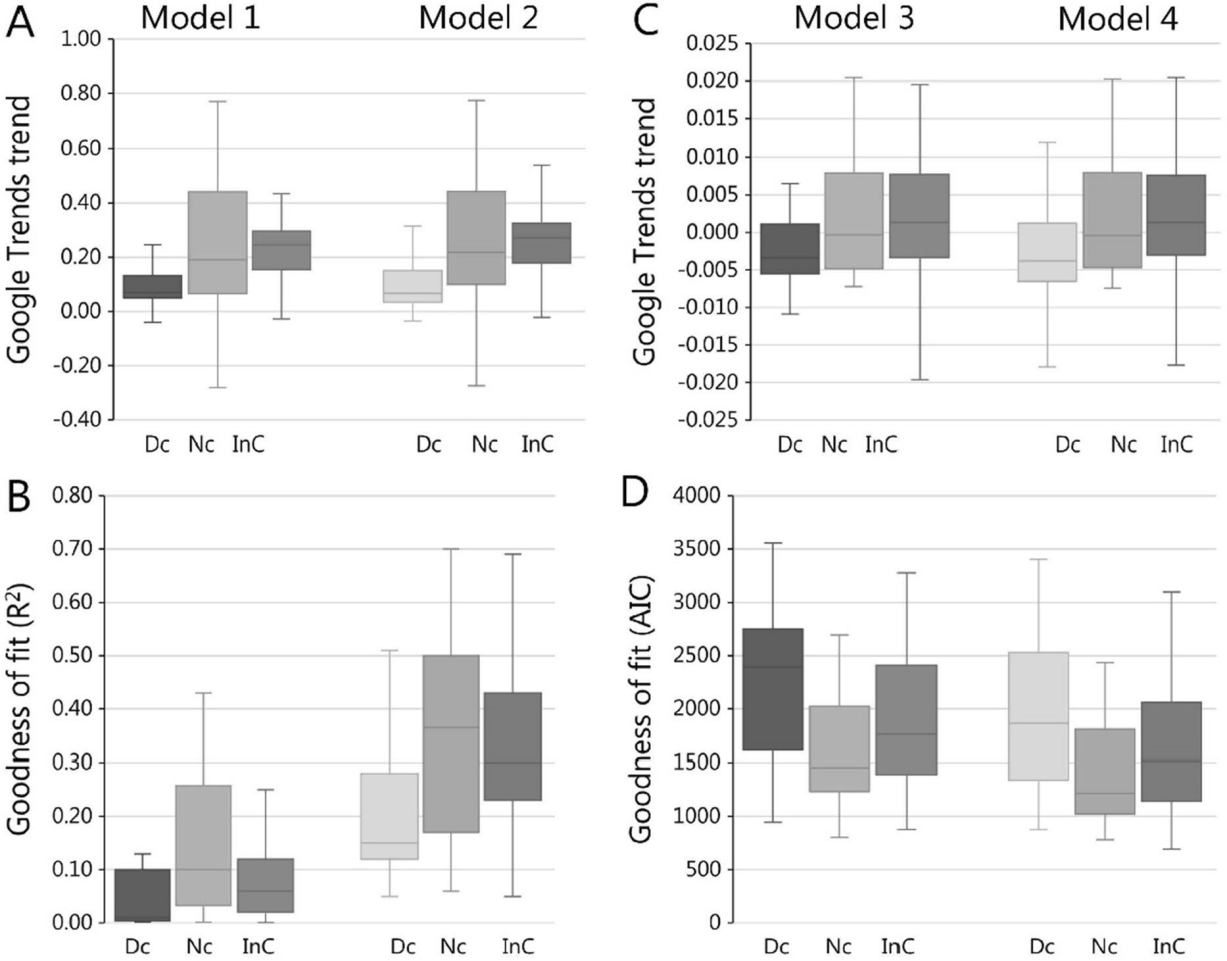
The temporal trends for models 1 to 4. **A** Trends for models 1 and 2: the change in GT frequency per month and **B** the associated goodness of fit for the 106 species (*R*^2^). **C** Trends for models 3 and 4: the change in contribution relative to the sum of GT frequencies for 129 species and the associated goodness of fit (AIC). The outcomes are given for species that by Danish Birdlife (DOF) were assessed to be in the declining (Dc), no-change (Nc), or increasing (InC) category

The temporal GT trends were lower for species that according to DOF were in decline in all four statistical models (Table 1). In models 1 and 2, all trends were positive, but the increments for declining species were half that observed for no-change and increasing species (Fig. 4A). For models 3 and 4, negative trends were observed for species in decline, the trend was unclear for the not-changing species, while there was a positive trend for increasing populations (Fig. 4C). The variation over the mean trend was however substantial (Fig. 4), and there was no statistical difference in the GT trends for not-changing and increasing species in models 1 and 2 (Table 1). Neither were there any statistical differences between declining and not-changing species in models 3 and 4.

**Table 1:** Mean Google Trends trends for species which by DOF was categorized as declining (− 1), not-changing (0), or increasing (+ 1). *p* values signify whether the means are different from zero, and the *t* test annotation informs whether the three means differed. Letters a and b signify mean GT trends that are not statistically different. Models 1 and 2: the change in GT frequency per month, and models 3 and 4: the change in contribution to the sum of GT frequencies for 129 birds

Model	DBL trend	Mean	SE	*t* value	*p* value	*t* test
Model 1	− 1	0.10	0.04	2.5	0.014	a
	0	0.21	0.04	5.32	< .0001	b
	1	0.22	0.02	9.85	< .0001	b
Model 2	− 1	0.10	0.04	2.58	0.011	a
	0	0.22	0.04	5.49	< .0001	b
	1	0.24	0.02	10.61	< .0001	b
Model 3	− 1	− 0.003	0.002	− 1.75	0.083	a
	0	− 0.002	0.002	− 0.93	0.357	a
	1	0.002	0.001	1.75	0.083	b
Model 4	− 1	− 0.004	0.002	− 1.99	0.049	a
	0	− 0.002	0.002	− 1	0.319	a
	1	0.002	0.001	1.91	0.059	b

### Assessing the agreement between observers

When assigning GT trends for model 2 to categories such that trends less than 0.1 were accepted as declining, GT trends from 0.1 to 0.2 as not-changing, and trends larger than 0.2 were increasing, we identified 31 species in the declining, 22 in the not-changing, and 53 in the increasing category. This agreed with the DOF categories for 56% (59/106) of the species (Table 2). The agreement for the remaining models was less, and the weighted kappa ranged from 0.20 to 0.33, i.e., fair agreement. Models 1 and 2 had a higher agreement with the DOF categories than models 3 and 4, indicating that the actual search frequencies had higher agreement than the relative search frequencies (Table 2). The trends in the two types of models (1 and 2 vs 3 and 4) were however quite similar (Pearson’s correlation coefficients > 0.85, *n* = 106, *p* < 0.001).

**Table 2:** Overview of the statistical assessment for the agreement between DOF trends and Google Trends trends for four different statistical models. The cutoff values were used to assign categorical trends for the GT data, e.g., such that trends less than 0.1 in model 1 were accepted as declining, GT trends from 0.1 to 0.2 as not-changing, while trends larger than 0.2 were increasing. Kappa values are weighted kappa except for the 2 × 2 table. (A) 27 larger species (wingspan > 100 cm) removed. (B) A further nine species mentioned in the cookbook were removed. (C) Forcing the individual record to be either − 1 or + 1, by narrowing the cutoffs. (D) Forcing GT trends into being either − 1 or + 1 and removing species that in the DOF records were given as no-change

Model	Data included	Cutoff values		Kappa	SE	95% CL		No. of agreements	Total obs	%
Model 1	All	0.1	0.2	0.33	0.08	0.17	0.48	58	106	55%
Model 2	All	0.1	0.2	0.32	0.08	0.16	0.48	59	106	56%
Model 3	All	− 0.005	0	0.20	0.08	0.06	0.35	53	106	50%
Model 4	All	− 0.005	0	0.23	0.08	0.07	0.38	54	106	51%
Model 2	No large species (A)	0.1	0.2	0.41	0.09	0.23	0.59	48	79	61%
	No recipes (B)	0.1	0.2	0.43	0.10	0.23	0.62	44	70	63%
	Narrow cutoff (C)	0.15	0.175	0.45	0.10	0.26	0.64	46	70	66%
	2 × 2 Table (D)	0.175		0.54	0.12	0.31	0.77	45	57	79%

### Identification and removal of species with lessor agreement

The disagreement between GT and DOF trends was mainly connected to species populations, which by either DOF or GT trends were noted as not-changing (Table 3). Larger birds were prominent in this group, and the removal of 27 larger species (wingspan > 100 cm) led to substantial agreement (61%, 48/79) for model 2. Removing a further nine species that were mentioned in an online Danish cookbook increased the agreement to 63% (44/70). Narrowing the cutoff values, and reducing the number of no-change species, increased the agreement to 66% (46/70). Deleting all species which by DOF had no-change populations and setting a single cutoff at 0.175 between declining and increasing populations delivered an agreement of 79% in a 2 × 2 frequency Table (45/57; Table 3). It therefore seems the general reliability of GT trends is significantly affected by species that due to their biological and culinary attributes receive varying degrees of interest.

**Table 3:** Frequency table for model 2 estimates as generated by the entire data set (*n* = 106) and wide (0.1 and 0.2) cutoff values that generate 13 species in the no-change group, and a narrowly defined set of species (*n* = 70) with a cutoff that depletes the number of species in the no-change group

		GT Trend			Agreement (%)
All		− 1	0	1	
DOF trend	− 1	14	3	4	
	0	5	6	10	59 of 106
	1	12	13	39	(56%)
Narrow cutoff					
DOF trend	− 1	13	1	4	
	0	4	2	7	46 of 70
	1	7	1	31	(66%)

### The outcome weighed against the challenge

Detecting population trends in animal populations is difficult due to uncertainties in abundance and range estimates (Krebs, 1999, Seber and Schofield, 2019). The uncertainties negatively affect our ability to accurately determine minor changes within short(er) periods. The trends may also be difficult to assess when a species decreases in abundance but at the same time expands its range, e.g., the Cormorant (*Phalacrocorax carbo*). Reaching a firm conclusion for a population that multiplies 5 to tenfold over a breeding season and suffers a substantial reduction in the winter may not yield clear population trends, which is further complicated by the method by which the “trend” is determined. It is therefore quite remarkable that the results show fair agreement between the trends in population development for the majority of Danish bird species. The lack of agreement connects primarily to populations that by DOF are assigned to the no-change category, as these often are considered as increasing in the analysis of the GT data.

We suspect that perturbations due to COVID-19 lockdowns in 2020 and 2021 may have inflated the GT observations (Brock et al., 2021), leading to elevated trends for some populations. It is also conceivable that Danish googlers suffer a bias connected to the size of the birds since the agreement increases when larger birds are removed. Negative trends may be caused by a loss of interest, as several, once rare, larger birds (e.g., white-tailed sea eagle (*Haliaeetus albicilla*), common crane (*Grus grus*)) now are fairly common. Inversely, the removal of species that appear in cookbook recipes (pheasants (*Phasianus colchicus*), partridges (*Perdix perdix*), and mallards (*Anas platyrhynchos*)) improves the agreement because the search for recipes induces a false indication of stability. Beyond these problematic populations, we suspect that the trends in urban areas could differ from the general national trend.

With a more than fair agreement, it follows that the two sets of observers also would agree on some of the ecological challenges that the Danish bird populations are facing. The DOF records presented in Fig. 3 show that rare species with limited range have declining populations, while more commonly occurring birds often have increasing populations. This development will lead to “homogenization of the bird community” (Clavero & Brotons, 2010; Devictor et al., 2008), which over time reduces the bird diversity. We suspect that Danish birds are affected by agricultural activities that promote habitat homogenization and landscape fragmentation (Endenburg et al., 2019). Importantly, the GT data support the same deduction.

### Towards an early warning monitoring system

**Fig. 5:**
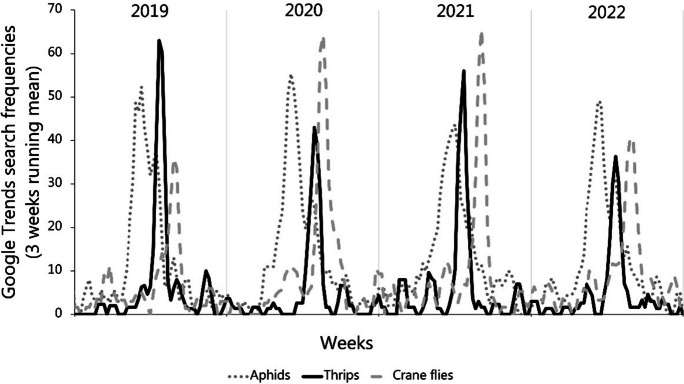
GT time series covering 2019 to 2022 (smoothed by 3-week running mean) for three different insects with distinct seasonal occurrence: aphids (Danish: bladlus), thrips (Danish: tordenfluer), and crane flies (Danish: stankelben). A disruption of the seasonal sequence would suggest an uneven change in temperatures in the micro-habitats they inhabit

**Fig. 6:**
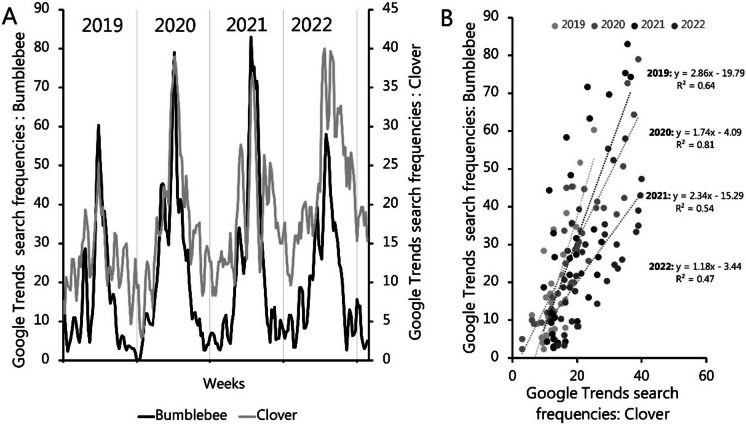
(**A**) GT time series covering 2019 to 2022 (smoothed by 3-week running mean) for bumblebee (Danish: humlebier) and clover (Danish: kløver). (**B**) Bumblebees vs Clover for week 1 to 35 i.e., until the bumblebee ceases to be active in late August

Online search frequencies may have a stronger positive correlation with population trends for birds than it does for other groups of organisms (Mittermeier et al., 2021) because our affinity to birds exceeds that of other animals. Yet, the high agreement between DOF volunteers and the Danish population will qualify the notion that the Danes provide credible information on trends in other animal populations as well. Thus, GT data could serve as a source of information on species that are not yet monitored by scientists, the government, or NGOs (Danielsen et al., 2009, Fig. 1). Such monitoring will like other types of monitoring permit us to detect changes in activity patterns for groups of invertebrates with widely different temperature sensitivities. But more importantly, it will allow for structured monitoring of relationships between organisms. Thus, the scope of the GT data allows us to combine “big data” with ecological knowledge (Jarić et al., 2020), and produce a wide array of early warning indices. We could, for instance, monitor the stability of the seasonal activity periods for invertebrates such as aphids, thrips, and crane flies, and inspect whether the peak in thrips remains between the peak of aphids and crane flies (Figures. 5 and 6). We could also incorporate information on plants because flowering plants attract more attention, which leads to seasonally varying frequencies (Fig. 6). In exploiting these indices, we can widen the scope and assess the specific relationship between certain plants and pollinators such as bumblebees and clover (Fig. 6). The two examples (Figs. 5 and 6) illustrate that we can translate environmental change into biological metrics and consider interspecies relationships such as the varying correspondence between flowering plants and their pollinators.

Acceptable credibility combined with unrivaled scope suggests that GT-based monitoring and other big data platforms (Johnson et al., 2023) by their versatility have significant potential for early warning monitoring of biodiversity. In our view, such a system would be analog to the tsunami-warning system in the Pacific Ocean—i.e., thousands of individual buoys, which permit us to measure subtle but widespread declines among multiple taxa that will pose a threat to biodiversity and ecological function. It is also conceivable that we can monitor “systematic perturbations” which precede species decline. GT can deliver the required data coverage, but it will take specialists to sieve through the data records and identify appropriate “buoys” that are capable of delivering reliable information.

## Conclusion

We find that the search frequencies provided by the Danes correspond well with the bird population trends observed by DOF, and we propose that the Danes have a similar ability for other types of animals. Early warning of biodiversity decline due to, e.g., climate change will likely be reflected in the GT records provided by the Danish population.

## Data Availability

The data for this study can be downloaded from Google Trends and information on Atlas III from DOF.
